# Guidance by physicians and pharmacists during antidepressant therapy: patients’ needs and suggestions for improvement

**DOI:** 10.1186/s12888-017-1522-9

**Published:** 2017-12-04

**Authors:** Mariёtte Nederlof, Daniёlle C. Cath, Lennart J. Stoker, Toine C. G. Egberts, Eibert R. Heerdink

**Affiliations:** 1 Division of Pharmacoepidemiology and Clinical Pharmacology, Utrecht Institute for Pharmaceutical Sciences, Utrecht University, P.O. Box 80082, 3508 TB Utrecht, The Netherlands; 2 Clinical Pharmacy, Brocacef Ziekenhuisfarmacie, 3600 AB Maarssen, The Netherlands; 3 Department of Medical Specialist Training, Drenthe Mental Health Institute, 9404 LA Assen, The Netherlands; 4 Department of Psychiatry, University Medical Center Groningen, 9713 GZ Groningen, The Netherlands; 5 Clinical Pharmacy, University Medical Center Utrecht, 3508 GA Utrecht, The Netherlands; 6 Research Group Innovation of Pharmaceutical Care, University of Applied Sciences Utrecht, 3508 AD Utrecht, The Netherlands

**Keywords:** Qualitative research, Focus groups, Antidepressiva agents, Second-generation, Counseling, Patient satisfaction, Professional role

## Abstract

**Background:**

Guidance of patients treated with antidepressants is paramount for successful therapy. The aim was to assess patients’ needs and suggestions for improvement of guidance by physicians and pharmacists during second generation antidepressant (SGA) therapy.

**Methods:**

Five focus group discussions were held with a total of 34 patients using an SGA. The discussions were conducted flexibly and responsively using a semi-structured topic list. All focus group discussions were video-recorded and transcripts were analyzed using ATLAS.ti for coding, thematic and open analysis.

**Results:**

Participants stated they were in need of better guidance. They suggested improving content of information during decisional moments, patient-health care professional communication and communication between health care professionals, and finally, organization of guidance. Barriers to achieving improved guidance were cited.

**Conclusions:**

Content, communication and organization of guidance are pivotal for achieving optimal guidance. Participants mentioned their current experienced guidance had limitations and brought up solutions for improvement. A next step would be to discuss the suggested solutions with health care professionals to assess their views and to discuss the possibility for implementation. After implementation, future studies could be aimed at determination of its impact on patients’ treatment efficacy, quality of life, treatment satisfaction and healthcare costs.

## Background

**Fig. 1:**
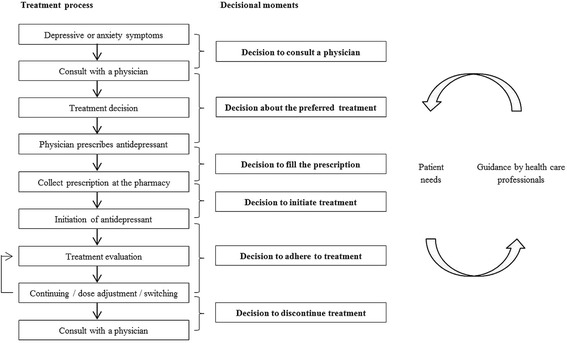
Decisional moments before and during antidepressant use

Antidepressant prescribing has increased significantly over the last decades [1–3]. There are concerns about over- as well as underdiagnosing patients and about under- and overprescribing of antidepressants [4]. Health care professionals focus on a correct diagnosis in patients with a major depressive or anxiety disorder, choosing a suitable treatment strategy and guiding patients. Patients need monitoring and guidance at decisional moments regarding treatment (Fig. 1). Shared decision making, incorporating patients’ needs is recommended [5, 6] and demands that both patient and health care professional are involved, share information, reach consensus about preferred treatment and implementation of treatment [7].

The effectiveness of treatment with antidepressants is influenced by selecting optimal medication, guidance of health care professionals and adherence of the patient to treatment [8]. Between 40% and 70% of patients with a major depressive or anxiety disorder fail to respond to second generation antidepressants (SGA) and up to 90% experience side effects [9–11]. Patients are facing multiple decisions before and during treatment that can influence adherence [12]. Approximately one in four patients receiving a first-time prescription for an antidepressant either do not fill the prescription at all (4%) or do not persist in use for longer than two weeks (24%) [12]. In addition, more than half of patients discontinue within six months [13, 14].

Half of the patients who do not initiate SGA treatment do so due to fear of side effects [15]. The decision to discontinue medication early can be a result of limited guidance by health care professionals during treatment, lack of knowledge and use, a negative attitude towards SGA use and the disease itself [15]. Patients who discontinue treatment early, perceive counselling by general practitioner and pharmacist as limited, both during initiation and execution of treatment [15]. Lack of guidance may also lead to unnecessary long duration of treatment.

In order to provide optimal guidance, the experiences and views of patients should be known and taken into account. Therefore, the objective of this study is to assess patients’ needs and suggestions for improvement of guidance by physicians and pharmacists during SGA therapy.

## Methods

The Institutional Review Board of the Division of Pharmacoepidemiology and Clinical Pharmacology of Utrecht University and the Research Committee of Altrecht Mental Healthcare reviewed and approved the study protocol. All participants provided written informed consent.

A qualitative approach was used to assess patients’ perspectives. Focus group discussions were held to assess experiences with regard to guidance during SGA therapy in-depth and to include the interaction with other participants.

To recruit patients, approximately 2500 brochures were dispensed in 40 community pharmacies from the Utrecht Pharmacy Practice network for Education and Research (UPPER) [16]. To include a diverse patient population, patients were also recruited at Altrecht Academic Anxiety Centre, Utrecht (a specialized mental health institution on secondary and tertiary treatment of anxiety disordered patients). Patients aged ≥18 years with a prescription for a second-generation antidepressant (Appendix) in the last twelve months were eligible. The participants did not know the researchers who conducted the focus groups before inclusion.

Focus group discussions were held in a private room for approximately two hours each, with six to eight participants per group. One female researcher (MN, PharmD), working additionally as pharmacist, conducted all focus group discussions using a semi-structured topic list, with a female psychologist as research assistant (NF) and a male second researcher as observer (EH, PhD). To gain experience, the primary author participated in a qualitative research training course. Thereafter, a pilot focus group was held to practice and improve the focus group setting. The objective of the study was explained to participants before start of the focus groups.

Group discussions were conducted flexibly and responsively, to enable participants’ narratives to develop. Participants were asked to write down two positive and two negative aspects of the guidance during antidepressant therapy. These notes served as basis for a brainstorm on recommendations for improvement of guidance by physicians and pharmacists. Participants were asked how they were guided during antidepressant therapy and about their opinion of received guidance using open-ended questions. Thereafter, it was discussed how guidance could be improved. Needs and suggestions for improvement of participants were summarized and presented to cross check their perspective. The number of focus group discussions was judged sufficient if data saturation was reached, discussed by the researchers after every focus group (MN and EH).

All focus group discussions were video-recorded and written down in their original language ad verbatim, to preserve the participants’ original meanings. Transcripts were imported and analyzed using ATLAS.ti (Version 7.5; Scientific Software Development GmbH, Berlin, Germany). Data were analyzed using an inductive data-driven process known as the grounded theory [17], a method of using empirical data without preconceived theories. The first level of data analysis consisted of a thematic analysis and the second of an open analysis of all transcripts by the first author (MN). A second author (EH) reviewed this analysis. Identified codes were arranged into meaningful themes and adjusted in a cyclic process with close involvement of the second investigator (EH). Although the data have not been discussed with the participants, they can be in future after translation in their original language.

## Results

Five focus group discussions included a total of 34 patients using SGAs (Table 1). During focus group discussions, multiple needs, barriers and suggestions for improvement of guidance were mentioned. At analysis, themes emerged; content of guidance, communication aspects and organization of guidance. Communication aspects were divided into communication between patient and health care professional and communication between health care professionals.

**Table 1:** Characteristics of focus group participants

Characteristics	N	%
Gender		
Male	7	21
Female	27	79
Age range		
18–29	7	21
30–39	6	18
40–49	7	21
50–59	6	18
> 60	8	24
Used antidepressant(s)		
Paroxetine	13	38
Venlafaxine	9	26
Sertraline	4	12
Mirtazapine	3	9
Citalopram	2	6
Escitalopram	2	6
Fluoxetine	2	6
Fluvoxamine	2	6
Therapy status		
Current user	31	91
Past user	3	9
Initial prescriber		
Psychiatrist	21	62
General practitioner	13	38
Self-reported indication		
Depression	15	44
Anxiety	5	15
Anxiety and depression	12	35
Other	2	6
Duration of SGA usage		
0–6 months	3	9
6 months – 1 year	4	12
1–5 years	11	32
5–10 years	7	21
> 10 years	9	26

### Content of guidance

Participants wanted guidance not only to focus on medication, but also on cause of symptoms and non-pharmacological therapy. Some felt that general practitioners prescribed an SGA too rapidly. Information on what medication participants were given, why this medication was chosen, discussing alternative treatment options, mechanism of action, assessment of harm-benefit balance and involving the patient in the decision-making process was needed.

*Woman, 36 years: “They only assess how to solve the symptoms of the depression, but not the cause.”*

*Male, 39 years: “Another thing I worry about, is that there are so many different antidepressants and they never told me why I get this one drug.”*

Incorporating a waiting time before initiation of the SGA was suggested to consider the decision to initiate treatment. A protocol for physicians could help to select the most accurate medication and to discuss information needed by patients. Participants noticed that it differed per physician what medication you receive; to the participants it seemed random. To help choose the right medication, patient questions could be included, for example to assess which symptoms are most prevalent for that specific patient. Monitoring of possible side effects or laboratory values was recommended and made participants feel more supported.

*Woman, 26 years: “Maybe a kind of protocol about how guidance should be. I have the feeling that it differs a lot between different physicians and that you have to be lucky.”*

Information needed to be provided on expected effects, side effects, difficulty of discontinuing treatment and potential worsening of symptoms during start of treatment. Some patients had doubts if they would have started treatment if they had been informed on all side effects; others felt incapable of making the choice to initiate treatment due to the severity of their symptoms. Patient leaflets were not always handed to patients in psychiatric institutions.

*Male, 63 years: “They put something in your mouth in a psychiatric institution, but you have no idea what it is.”*

*Woman, 26 years: “The physician could also mention that if you experience these side effects, you have to contact him. The side effects are of course listed in the patient information leaflet, but I think it is important that the physician mentions them.”*

Subjects that a physician should discuss during treatment were worries, side effects, complaints, the patients’ wellbeing, considering early discontinuation, utilization of and experience with medication, the patient’s social network and suicidality. If needed, physicians should discuss the possibility of switching medication. The efficacy of the medication, complaints, side effects and patients’ worries should be discussed by the pharmacist too.

*Woman, 45 years: “I would like to be monitored for the side effects that I experience. How bothersome are the side effects for me? What is the effect on my body? Is there a safer medication or should the dosage be adjusted?”*

For patients, it would be helpful to have a guideline to self-evaluate efficacy, dose and side effects of medication. It was difficult to evaluate efficacy as mood can vary or to determine if a side effect was related to the antidepressant. An evaluation form for patients was proposed to assess all side effects, including sexual side effects. This could help less assertive patients to formulate questions preceding consultation. The physician could then provide feedback on patients’ questions.

*Woman, 34 years: “It can be a standard question to fill in a form before your six-month-control. You can write down how you feel and what your worries are.”*

*Woman, 52 years: “I would like to fill out such a form at initiation, after four weeks and so on to discuss side effects. Are my side effects due to the medication or due to something else?”*

In case of long-term treatment, the physician should assess and advice if it is necessary to continue, discontinue or adjust the dosage of medication. Guidance during long-term use, dose adjustment and after cessation should be available and based on practical tips for dose reduction and by discussing the patient’s wellbeing and experiences. The proposed protocol could include the requirement to inform patients about treatment discontinuation and methods to do so. Consultation after tapering was recommended.

*Woman, 67 years: “You receive the medication and are thrown in the deep end. Do I take it for a year or ten years or endless? You don’t know, but I do not dare to cease treatment.”*

### Communication aspects

#### Communication between patient and health care professional

Participants mentioned the need to be taken seriously most often. Participants felt that not all psychiatrists were empathic enough, especially in case of side effects or during initiation or tapering off medication. Direct contact, accuracy, respect and an attitude of thinking along with the patient were essential.

*Woman, 29 years: “I was very depressed but my psychiatrist was not empathic during initiation of medication. At a moment I was unable to carry on with the increase in dosage, because I had such severe side effects. He mentioned: “You are 26 years old, behave like an adult.” In the end that approach worked, but I thought it was harsh.”*

The autonomy of patients should be respected. Shared decision making about dosage or choice of antidepressant was experienced positively, while other participants stressed the importance of being able to make their own choices, based on good advice.

*Male, 48 years: “Yes, I am the boss beyond any doubt. My internist is my manager.”*

Mutual trust between physician and patient was cited as being important for medication initiation and believe in efficacy of treatment. Participants did not completely trust their physician if they could not recall conversations, medication dosages, gave opposing advice, made mistakes in prescriptions or were chaotic. Some participants liked the ability to choose for a female or male physician.

*Woman, 52 years: “I can send an email to my psychiatrist if I think the dosage needs to be adjusted and why. Then I can receive the medication because he trusts me, he knows that I know my body; I know when things go wrong, in the direction of a depression. And I think that’s fantastic.”*

Hierarchy was noticeable for some participants and made it difficult for them to discuss questions. Equality was needed in verbal and non-verbal communication.

*Male, 63 years: “I did not get to see a psychiatrist, only on the background the psychiatrist decided what to do, evaluated how I acted and which medication to choose. It confused me. In another hospital communication was better and reports were made every two weeks of my wellbeing.”*

#### Communication between health care professionals

Communication between health care professionals was seen as insufficient when advice was conflicting or transfer of information was missing on the patients’ medical history. Some participants felt that communication between health care specialists in psychiatric institutions was better organized. It was sometimes unclear for participants which health care professional to contact for guidance.

*Male, 61 years: “What I consider to be negative is that psychiatrists establish different diagnoses. For years I have taken a blue pill because I had attention deficit hyperactivity disorder (ADHD). Until the next psychiatrist said “That man does not have ADHD” and I had to quit immediately.”*

*Male, 49 years: “I was often referred by the general practitioner to the psychiatrist and the other way around. But in between I was left to my fate and I still am.”*

Participants preferred to have a single health care professional responsible, to keep track of treatment processes. That health care professional should assess if treatment is still adequate with respect to dosage, duration of use and the patients’ wellbeing. However, it was difficult to decide who this responsible health care professional should be.

*Woman, 25 years: “I was 17 years old when I got the prescription. My general practitioner was a bit hesitant to prescribe because he thought I was young, but after I called the psychiatrist I got the prescription. I have also been to different clinics and every time another person took over the guidance as a consequence. What I have missed is a kind of overall responsibility. Who is responsible and keeps an eye on me?”*

### Organization of guidance

The preferred frequency of contact differed between participants, but in general they felt it should be intensified. Some participants mentioned that their physician contacted them only once or a few times. Others lost contact after long-term use of the medication.

*Male, 39 years: “Even when I was admitted on a closed unit of a psychiatric hospital, I rarely got to see a psychiatrist.”*

*Woman, 64 years: “My general practitioner prescribed the medication, there was no follow up afterwards. I can request repeat prescriptions. I think it is wrong that he never asks about it or that there is no such thing as a short conversation.”*

Participants explained that when you have a depression or anxiety disorder you are inclined to retreat and avoid contact. Periodical consultations during initiation of treatment were needed and after every consultation it should be clear when the next appointment is. Initiative for contact should come from the physician, since not all patients are assertive enough. Preferred frequency of contact was once to every week during the first month and monthly to every six months during maintenance therapy. Further, duration of consults was perceived as insufficient.

*Woman, 47 years: “Only recognizing your feelings, by proposing to set appointments every six weeks, or to call at a fixed time once a month, whatever you want. If a physician suggests that, it will already be part of the solution, you then know it is not going to be easy and someone is there for me.”*

*Woman, 45 years: “The psychiatrist should monitor, explain the mechanism of action and side effects, should take care of a follow-up appointment and is responsible to keep the general practitioner updated about the use of that medication.”*

Some participants mentioned that they should preferably be informed about SGA therapy with a relative, friend, neighbor or somebody who is close to the patient present. Patients should not initiate an antidepressant without a relative or caregiver monitoring them.

*Woman, 36 years: “I honestly think a patient should not be left alone during the first day of medication initiation. It can even be your mother or a friend who keeps an eye on you.”*

*Woman, 29 years: “I totally agree. I had suicidal thoughts during the first five days”.*

It was sometimes difficult to reach a psychiatrist when needed, for example when experiencing suicidal thoughts or psychotic symptoms, side effects at night, during weekends or during a medication switch.

*Woman, 59 years: “During the evenings and in the weekends, there is no one and especially in those times things are heading your way. I think that is a disadvantage.”*

In general, contact with pharmacists was limited. It was appreciated if pharmacists explained the choice of medication, asked about side effects, checked for medication interactions, gave a medication review or supported with SGA tapering. Privacy in pharmacies was perceived as inadequate; therefore, some participants preferred to phone about questions. Participants thought it was frustrating when medication was out of stock, when brands changed frequently and costs for dispensing were thought to be high.

*Woman, 27 years: “I always speak to a pharmacy assistant and I think it is not substantial enough. To me pharmacists are primarily for the distribution of medication.”*

Some participants wondered why they could fill in repeat prescriptions, even through the Internet, for a long time without a check. A participant mentioned that an alert to pick up repeat medication would be appreciated and suggested to contact a patient if the prescription was not filled.

*Woman, 26 years: “For repeat medication you have to keep in mind that you should be in time. Once I was very stressed because I was too late and you can get withdrawal symptoms if you do not receive the medication in time. It could possibly be improved with announcements.”*

A daily consultation hour by the physician or pharmacist was mentioned as a solution to increase contact, this could even be by email or phone, and needed to be provided within psychiatric institutions too. A stand-by 24-h service helpline for patients in need was mentioned, to be able to ask questions, receive information and feel heard. When available, awareness for that possibility should be created.

*Woman, 28 years: “That people feel heard and do not have to wait for a week. Especially during dose increases or ceasing a drug, because that can cause serious reactions such as suicidal thoughts and that just can’t wait.”*

Participants had mixed impressions on use of eHealth solutions for guidance, such as a mobile application. Some worried that contact would become too frequent, it would be too busy for health care professionals or had no smartphone. An anonymous private forum with a chat function, monitored by a psychiatrist was mentioned, although reading other experiences could possibly make participants anxious. Contact with health care professionals by email was appreciated.

*Woman, 36 years: “I would prefer just face-to-face contact.”*

*Woman, 45 years: “It would be a good aid to keep and at least improve contact, to be able to communicate experiences, to improve monitoring.”*

Some participants were of opinion that only psychiatrists should prescribe antidepressants because of a perceived limited knowledge of general practitioners on side effects, alternative medication and medication interactions. Knowledge of pharmacists was also thought to be limited. Reasons not to have a psychiatrist as prescriber were related to a better relationship with the general practitioner or because participants did not want more consultations with the psychiatrist.

*Man, 63 years: “If I feel worse again, I would go to the psychiatrist. I do not have trust that the general practitioner is appropriately qualified.”*

*Male, 49 years: “You go with side effects to the general practitioner, you mention them and then you have to choose. What medication do you want?”*

*Woman, 28 years: “Yes that is what you are being asked, as if you are the expert!”*

## Discussion

Participants reported their current experienced guidance had limitations and brought up solutions for improvement and potential (already experienced) barriers. Consistent with previous work, we found content to be important for satisfaction with a consultation [18]. Counselling is not a single event, but a process over time [19, 20]. Providing treatment expectations and knowledge may improve adherence and treatment success [21, 22]. Our study showed that patients were in need of information throughout treatment for decision making, at start of treatment, during continuation and at end of treatment. Clear advice during decisional moments was essential, including what to expect regarding effects and side effects.

Shared decision making, involving patients in treatment decisions and incorporating patients’ preferences, was of major importance, which is in agreement with other (qualitative) studies [21, 23–25].

Previously, patients have emphasized the importance of communication and behavior of health care professionals, rather than the amount of time the practitioner provided [6]. Percival et al. reported that patients felt like they were given sufficient time when practitioners focused attentiveness. Our study emphasizes the importance of communicational aspects such as approachability, empathy, support and active listening. Contrary to Percival et al. participants in our qualitative study mentioned a clear need for intensified contact. A higher frequency and longer duration of consultations were suggested. Not only communication with the patients, but also between health care professionals should be improved.

Needs of patients were explored in-depth and participants provided suggestions on how to improve guidance. This knowledge is needed to improve guidance in clinical practice.

The sample may not have been a cross-section of SGA users. Patients who have a strong opinion about their received guidance will possibly be more inclined to contribute to focus group discussions. Therefore, it is uncertain if the results are generalizable. Our patient selection consisted of patients who succeeded to initiate treatment and adhered to treatment for at least six weeks, with more than half of the participants on SGA treatment for more than six months. Some participants used SGA’s for over ten years and might not have remembered guidance during start of treatment precisely, which could have led to recall bias.

A next step would be to discuss suggested solutions with health care professionals to assess their views and to discuss the possibility for implementation. Future studies could be aimed at determination of its impact on patients’ treatment efficacy, quality of life, treatment satisfaction and healthcare costs.

## Conclusions

Participants were in need of better guidance. Although improvement of guidance in clinical practice is comprehensive and complex, our study provides recommendations from patients themselves on needs and how to improve guidance, which are highlighted in Table 2. Content, communication and organization of guidance are pivotal for achieving optimal guidance. Participants additionally reported potential (already experienced) barriers. The recommendations from patients can aid health care professionals to improve guidance in clinical practice.

**Table 2:** Patients’ needs, suggestions and cited barriers for improvement on guidance by health care professionals

Patient needs	Suggestions for improvement of guidance by health care professionals	Barriers
Content of guidance		
Before the decision to fill a prescription		
Information needed on: - cause of symptoms - treatment options	Shared decision making, incorporating patients’ needs	Patients might not be capable of making a choice in case of severe symptoms
	Incorporate time before initiation of an antidepressant to consider the decision to initiate treatment	
The physician should choose the most accurate medication	Provide a protocol for physicians to help select the most accurate medication by using questions on disease symptoms of patients and to assess side effects and laboratory values	
During the decision to initiate SGA therapy		
Information needed on: - rationale behind drug choice - mechanism of action - harm/benefit balance - expected effects and side effects (also during discontinuation) - potential worsening of symptoms during start of treatment	Mention needed information explicitly	Patients might be reluctant to start therapy when knowing potential side effects
	Provide patient leaflets (also within psychiatric institutions)	
During the decision to adhere to or to discontinue treatment		
Health care professionals should discuss: - worries - side effects - complaints - the patients’ wellbeing - if the patient considers discontinuation - medication use and experiences - the patients’ social network - suicidal thoughts	Evaluate treatment with the patient	Limited time of a consultation
	Provide a guideline for patients to evaluate efficacy, dosage and side effects of the antidepressant	
	If necessary, discuss the possibility to switch treatment	
	Evaluation form or questionnaire to assess side effects together with the patient	
During the decision to discontinue treatment		
Guidance during long term antidepressant use is needed to assess if the dosage needs to be adjusted, and to decide to continue or discontinue treatment	Assess whether it is necessary to continue treatment and provide information on the expected treatment duration	Patients may change their dosage or discontinue treatment without informing their health care professional
	Guide the patient during long-term use and if the decision is to discontinue therapy, give practical tips on how to do so.	
Communication aspects		
Communication between the health care professional and the patient		
- take patients seriously - be empathic - provide direct contact - be accurate - respect the patient - attitude of thinking along - respect the autonomy of the patient	Carefully listen to the patients’ story	Some participants thought psychiatrists were not emphatic enough
	Provide a combination of a psychiatrist with a psychologist	
	Provide the ability to choose for a male of female health care professional	Some patients may not be assertive enough to request this
Mutual trust between the physician and patient	Keep a sound registration of information gathered in previous appointments	Some participants did not trust their physician if they could not recall conversations, medication dosages, gave opposing advice, made mistakes in prescriptions or were chaotic
Equality between health care professionals and patients	Show equality in posture and respect for the patient	
Communication between health care professionals		
Improvement of communication between health care specialists	Information should be transferred on the patients’ medical history and conflicting ideas between health care professionals should be solved to prevent confusion of the patient	
For the patient it should be clear which health care professional to consult	Assign a responsible health care specialist to keep track of a patient, assess if the treatment is still adequate and to assess wellbeing of the patient	It was not clear who the responsible health care specialist should be
Possibility to be admitted to a psychiatric hospital		
Organization of guidance		
Periodical visits during initiation and (long-term) treatment. It should be known when the next appointment is	Physician should be responsible for initiative of contact and discuss the preferred frequency with the patient	Patient might be not assertive enough to request a consultation
Longer duration of a consultation		
Involve social network during medication initiation	If wanted: involve social network during medication initiation. Physicians and pharmacists should advise not to initiate the antidepressant without a relative or caregiver monitoring them	Not all participants wanted to involve other persons in treatment or did not have a social network
Possibility to contact a psychiatrist when needed	Daily conversation hour, even by email or by phone, by the physician or pharmacist (also within psychiatric institutions)	Physician might not have time due to high workload
	Provide a stand-by 24-h helpline for patients in need	Inability of helpline operator to have insight into the patients’ medical file
	Provide an anonymous chat forum leaded by a psychiatrist and not visible for everyone	Reading other patients experiences can make patients anxious and others could write nonsense
More privacy in pharmacies	Provide more privacy in pharmacies	
Checks during repeat prescriptions	Provide an alert for repeat prescriptions	If a stock is inadequate, the patient might receive medication too late
	In case a prescription is not filled, call a patient to ask for the reason	
Health care professional should have enough knowledge on treatment	Only psychiatrists should prescribe antidepressants	Some patients have a better relation with their general practitioner
		Some participants did not wat to see their psychiatrists too frequently
	Knowledge of general practitioners and pharmacists should be improved	

## Appendix

**Table 3:** Included second generation antidepressants and ATC codes

Antidepressant	ATC code
Citalopram	N06AB04
Escitalopram	N06AB10
Fluvoxamine	N06AB08
Fluoxetine	N06AB03
Paroxetine	N06AB05
Sertraline	N06AB06
Venlafaxine	N06AX16
Mirtazapine	N06AX11
Duloxetine	N06AX21

## Abbreviations

SGA: Second generation antidepressant
